# Students' Experiences of Seeking Web-Based Animal Health Information at the Ontario Veterinary College: Exploratory Qualitative Study

**DOI:** 10.2196/13795

**Published:** 2019-11-08

**Authors:** Nanette Lai, Deep Khosa, Andria Jones-Bitton, Cate E Dewey

**Affiliations:** 1 Department of Population Medicine Ontario Veterinary College University of Guelph Guelph, ON Canada

**Keywords:** veterinary education, internet, computer literacy, focus groups, perception

## Abstract

**Background:**

Although searching for health information on the internet has offered clear benefits of rapid access to information for seekers such as patients, medical practitioners, and students, detrimental effects on seekers’ experiences have also been documented. Health information overload is one such side effect, where an information seeker receives excessive volumes of potentially useful health-related messages that cannot be processed in a timely manner. This phenomenon has been documented among medical professionals, with consequences that include impacts on patient care. Presently, the use of the internet for health-related information, and particularly animal health information, in veterinary students has received far less research attention.

**Objective:**

The purpose of this study was to explore veterinary students’ internet search experiences to understand how students perceived the nature of Web-based information and how these perceptions influence their information management.

**Methods:**

For this qualitative exploratory study, 5 separate focus groups and a single interview were conducted between June and October 2016 with a sample of 21 veterinary students in Ontario, Canada.

**Results:**

Thematic analysis of focus group transcripts demonstrated one overarching theme, *The Overwhelming Nature of the Internet*, depicted by two subthemes: *Volume and Type of Web-based Health Information* and *Processing, Managing, and Evaluating Information*.

**Conclusions:**

Integrating electronic health information literacy training into human health sciences students’ training has shown to have positive effects on information management skills. Given a recent Association of American Veterinary Medical Colleges report that considers health literacy as a professional competency, results of this study point to a direction for future research and for institutions to contemplate integrating information literacy skills in veterinary curricula. Specifically, we propose that the information literacy skills should include knowledge about access, retrieval, evaluation, and timely application of Web-based information.

## Introduction

### Seeking Health Information on the Internet

The phenomenon of health care consumers searching for health or medical information on the internet has been widely documented in the academic literature [1-3]. Furthermore, students enrolled in health care programs (eg, medicine, nursing, and dentistry) have been investigated extensively and are reported to use the internet for health-related information [4-6], for supporting their learning in junior years [7], and to help with their understanding of content during senior clinical years [8]. Presently, the use of the internet for health-related information, and particularly animal health information, in veterinary students has received far less research attention [9]. As a recent report from the Association of American Veterinary Medical Colleges (AAVMC) considers health literacy a professional competency [10], exploring students’ internet search experiences for animal health information may offer deeper insight about students’ digital health literacy.

### Health Information Overload

Although searching for health information on the internet has offered clear benefits of rapid access to information for seekers such as patients [11], medical practitioners [12], and medical students [13], detrimental effects on seekers’ experiences have also been documented [14]. One example of a negative outcome associated with Web-based health information seeking is so-called *cyberchondria* [15,16], where Web-based information seekers are reported to become overly concerned and anxious about their health signs and symptoms, and subsequently experience health information overload (HIO) [17-19].

Information overload (IO) involves having “access to more useful information than can be processed in a useful way” (p. 266) [20]. Similarly, HIO describes the phenomenon that occurs when an individual receives excessive amount of health-related messages such that potentially useful content becomes a burden rather than beneficial to the recipient [21]. Some researchers suggest that the internet is a major contributor to HIO in today’s society [14] with implications of potentially undesirable outcomes for information seekers [22]. For example, information seekers with HIO may develop a long-term unwillingness to seek information online, feel reluctant to further develop Web-based information search skills [23], or develop psychological effects such as anxiety [24,25].

Multiple studies suggest that HIO may be related to seeker health information literacy (HIL) [21,26]. HIL is defined as the degree to which individuals can obtain, process, understand, and communicate about health-related information needed to make informed health decisions [18,27]. HIL pertaining to health content delivered by means of the internet is termed electronic health information literacy (EHIL) [28,29]. Research suggests that academic institutions include EHIL training within their health care programs to address HIO potentiated by the internet [30].

HIO has been documented among human health care professionals [30,31], with potential impacts on patient care [32]. For example, Singh et al [33] reported physicians missing or overlooking patient test results as a consequence of being overloaded by a continual stream of information from internet-based electronic health records (EHRs). EHRs contain vast amount of medical content about individuals or populations, retrievable to stakeholders such as medical patients and providers [34]. The internet also contains other types of digital medical content accessible to health care professionals that are often used for training and educational purposes. Some researchers argue that students training in health care professions are also at risk of HIO [35]. Further supporting this concern are results from a survey of medical students [36], where 91% of the 100 respondents reported feeling overloaded by medical information. In addition, the same surveyed students indicated that they felt medical IO caused over 80% of their stress.

Stress among medical students has been well documented [37]. Similarly, stress, mental health, wellness, and their respective relationships to student life among veterinary students have also received research attention [38-43]. One qualitative study reported that upper-year Australian veterinary students’ primary source of stress came from IO [41], although information from the internet was not explicitly mentioned. Given that veterinary students today have ready access to the internet for animal health information [44], similar to their medical counterparts, veterinary students may also be at risk of HIO. Previous research shows that vast volumes of information may be a predisposing factor to HIO for postsecondary students with internet access [45] and potentially for veterinary students who reported searching for content using Google to support their studies [44]. Further research indicates that some veterinary students report their training as a chronic stressor [38], which Laakonen and Nevgi [39] suggest may arise from academic demands such as the overwhelming volumes of information students are expected to learn and assimilate. Moreover, HIO may negatively impact student learning as some researchers speculate IO reduces learners’ ability to process information, especially at a deep level [46].

To understand the effects of using Web-based health information, and the potential for HIO, one must first understand students’ experiences. The purpose of this study was to explore veterinary students’ Web-based animal health information search experiences. Specifically, we wanted to investigate how students perceived the nature of information generated from internet searches and how these perceptions influence students’ management of Web-based information.

## Methods

### Study Design and Participants

This exploratory qualitative study involved a series of 6 interviews with students enrolled in the 4-year Doctor of Veterinary Medicine program at the Ontario Veterinary College in Guelph, Ontario, Canada. All focus groups were conducted between July and September 2016. The study protocol was approved by the University of Guelph Research Ethics Board (REB #016AP002).

A total of 21 students were interviewed in 5 separate focus groups and a single individual interview. The mean number of participants for each focus group was 4 (range 3-5). Each focus group discussion ranged from 60 to 90 min. Students’ ages ranged from 21 to 34 years (median=23 years); 20 (95.2%) were female. At the time of data collection, participating students were enrolled in years 1 to 4 of the Doctor of Veterinary Medicine program.

### Recruitment

All student participants were recruited using an electronic mail listserv, physical posters displayed on bulletin boards located within school buildings, and snowball sampling. Snowball sampling involved requesting students to recommend peers or acquaintances who may also qualify for participation [47]. Students were informed of the study purpose and format and were offered an honorarium ($10 CAD gift card) and meal for participating. All participants were informed as to the risks, benefits, and repercussions of their involvement in the study, and accordingly consented to their involvement before the start of each focus group discussion.

### Data Collection

At the students’ convenience, interviews took place at the Ontario Veterinary College and followed a semistructured question guide (Multimedia Appendix 1) developed by the first and second authors to discuss the topics of: most recent internet searches for animal health information; internet resources accessed for investigating animal health information; challenges experienced when searching for animal health information on the internet; methods used by students for evaluating quality and validity of internet-based animal health information; and opinions about animal health information on the internet. Focus group questions were open-ended and designed to stimulate discourse among students. Before data collection, the question guide had been assessed using a pilot focus group consisting of a convenience sample of 3 graduate students at the University of Guelph.

Discussions between participants allowed the moderator (first author) to explore a range of perspectives and shared practices among students involving Web-based searches for animal health information. Data saturation [48] was achieved by the 6th interview as few insights emerge beyond 20 participants [49].

Field notes documenting observations and nonverbal behaviors were taken by the moderator during all focus group discussions. All discussions were audio recorded, transcribed verbatim, and deidentified to ensure that transcribed material could not be linked to individuals. Individual speakers were identified as S####. The prefix S indicates the speaker was a student, and #### consists of a unique number assigned to the student. Participants completed a short demographic questionnaire post interview to collect data on gender and age to describe the study population.

### Data Analysis

All transcripts were systematically checked against the audio recordings for accuracy of representation by the first author. The computer software QSR NVivo 11 was used to facilitate organization of transcripts for thematic analysis [50]. In brief, each transcript was read multiple times to facilitate familiarization of the data, and open codes were applied to sections of text illustrating common ideas across different student focus groups. Common codes occurring across groups were then organized into themes and subthemes and described in a codebook (Multimedia Appendix 2). The themes were then systematically reviewed, named, and defined. For consistency and clarity, naming and defining themes and subthemes were reviewed and cross-checked with codes by the second author. Any inconsistencies were discussed and resolved between the first and second authors. The third and fourth authors were responsible for approving the themes finalized by the first and second authors. Demographic data were analyzed using descriptive statistics (eg, means, median, and proportions) using Microsoft Excel.

## Results

### Theme: The Overwhelming Nature of the Internet

#### Subtheme: Volume and Type of Web-Based Health Information

A general perspective shared by students about seeking animal health information on the internet pertained to the volume of content they had encountered from past searches:

There’s just too much (information) out there.

S1453

Another student declared:

If I’m looking I sometimes get overwhelmed by the amount of information that is out there.

S1660

The students’ views about the overwhelming nature of animal health information online appeared to be related to the students’ search behaviors such as why they needed information.

#### Seeking Companion Animal Health Information

Expectedly, students searched on the internet because of concerns or questions about their personal companion animals’ health. These experiences reflect the students’ comments about being overwhelmed by large quantities of animal health information on the internet, as depicted by one participant:

[My search results] would list a whole bunch of symptoms that overlap with so many things. So, then you’re like my pet has this, and this, and this because everything kind of overlaps...so you’re not actually sure.

S1350

This statement also illuminated that amassing large volumes of content from the internet may confuse students.

#### Building Background Knowledge

Another reason driving students to the internet involved searching for foundational knowledge requiring exploration or understanding:

It’s a quick way to have a bit of a reference guide, or just background information on a topic that you’ve got no clue about.

S1658

Despite the speed with which foundational information could be retrieved, students agreed this increased the volume of content they needed to manage. This was best exemplified by one student’s comments:

Sometimes when we’re learning these new things...I don’t think it’s beneficial having all the resources...because there is so much more behind it that maybe we don’t understand...we have an end point but there’s so much back research behind it that you...need to know to understand and be able to explain the disease and why it happens. But you don’t have enough time to approach that.

S1245

This comment accentuated the problem of the internet facilitating students’ roundabout research efforts in navigating vast amounts of information because they felt that they *need to know to understand*. Similar to road traffic travelling through a circular intersection, students appeared to meander continuously in circles on the internet. These roundabout searches began with students gathering information to enhance their understanding, but the information collected also contained other ideas students were unfamiliar with. In attempting to clarify these other ideas, the students would then search for additional information. Ultimately, the students appeared to find themselves in a continuous research cycle that seemed to carry on infinitely, best described by one student:

It’s...an endless black hole of information that you can find out there.

S1660

Another student reported experiencing similar difficulties which began from being unable to recall the exact terminology depicting the desired information, owing to the limitations of previous knowledge:

You can’t find the original thing that the teachers threw out in class and you’re like I really want this...but you don’t remember how to write it and you look it up...and you can’t find anything that helps support what you’re trying to find.

S1244

#### Searching for Veterinary Information via Google

How students searched for information appeared to contribute to the students’ perceptions about large quantities of Web-based contents being overwhelming. In particular, almost all participants referenced using the Google search engine:

I’ll admit I started with a Google search on (a subject) and then weeded through the websites I thought seemed more legitimate.

S0628

Google searches also occurred within more specific settings such as during classes:

If...a professor says a word you don’t understand you can quickly Google the definition.

S1660

However, students quickly recognized multiple undesired consequence of searching on Google while attending lectures, as depicted by the following account:

I found so many really deep histo(logy) things...that doesn’t help. I found things that were basically just what (the professor) said on the slide and that doesn’t help either. I know the part I want is one step further but not five steps further... I couldn’t find something that was in-between to my own understanding.

S1352

As this participant’s words indicate, students were not only concerned about the volume of information Google may retrieve from the internet but were also encountering challenges in finding relevant information that correlated with what they required to enhance their understanding of a subject. However, students often found content appearing to contain levels of detail beyond what was needed for their stage of understanding.

Similarly, students recognized an undesired consequence of using Google to retrieve information:

[I] have a hard time narrowing down what you actually need to know and learn at this point.

S1660

Another participant shared a related belief, expressing the desire:

To know how to limit down the search to find exactly what you want.

S1244

Students clearly expressed a sense of being overwhelmed by the quantity of information generated by a Google search, and also shared experiences of frustration because the volumes of information generated was deemed irrelevant to their needs.

Beyond the Google search engine, students offered reasons as to why they may feel overwhelmed by the volume of information generated by a Web-based search, for example:

I think just some part of human nature...if you have a question you want the answer. And you don’t necessarily always want to sift through details.

S1661

Correspondingly, students unanimously expressed the desire to get immediate answers in a simple form rather than having to dig deeper through large volumes of detailed information.

#### Subtheme: Processing, Managing, and Evaluating Information

Related to students’ descriptions and efforts of acquiring large volumes of information, students across focus groups appeared weary when describing the need to further process their search results. One student specified that textual Web-based information presented personal challenges for the following reason:

I can only seriously read for so long and it’s just too much.

S1246

Similarly, a fellow student stated the following:

I have a really hard time looking at pages and pages of words.

S1661

Still another student shared experiences about encountering large volumes of text-based information on the internet, mentioning a specific medium:

Forums...I don’t like them at all. I try to stay away from forums...the thought for me reading through blog post upon blog post...I find maybe one word that will lead me to something else...just...really tedious. I find that...frustrating.

S1245

Another aspect students presented about their frustrations with internet content involved managing conflicting information, as demonstrated by one individual describing his search experience:

I would just go to the search bar, type in...eosinophilic granuloma complex, hit enter and then look at all these message board posts, which you spend hours ciphering through. Some people say treat with this, some people say treat with that. It’s just—it’s frustrating.

S1557

The apparent frustration expressed by students seemed related to becoming confused by Web-based content, which added another level of cognitive effort. This was depicted by one student’s comments:

Something like PetMD. Sometimes they can give you potential diagnoses...is this really reliable because it really fits all the symptoms that we were talking about? Or is it that...I’m just getting ahead of myself and it is listing a bunch of other things that it could be, that it really is?

S1349

#### Assessing Information Reliability

The student’s comment indicated that a large part of processing internet content entailed students evaluating information reliability.

Information processing and evaluation often involved students comparing internet information with their existing knowledge. For example:

I use my own background knowledge to decide whether...to read through it. And say...this sounds like a good information.

S1661

Using existing knowledge to compare against information found online appeared to be a common strategy among participants for discerning accuracy and validity of the information, as indicated by another student:

My roommate and I were finishing up a POD (Principles of Disease) assignment so we were looking up, we’re trying to differentiate between primary and secondary hemostasis and coagulation factors and stuff involved...things like that, where...I’ve learned it, and I have...a little bit of knowledge on it so I can...use that to help me guide whether or not...it’s truthful or not on the Internet.

S1453

This same student further explained her information assessment strategies:

It seemed to be the most reliable information just based on what knowledge I have already. It seemed relatively consistent with what we’re learning in therio(genology). And what I know just from past experience.

S1453

When the moderator asked the same student to describe her process referring to past experiences, the student further elaborated as follows:

I’ve worked for 4 years in a small animal practice, I volunteered for two years at large animal practices and I volunteered for five years at another small animal practice. Just comparing...cases we’ve seen at work...the outcome of the cases and the treatment protocol... then comparing that to what the forums and the websites would say.

S1453

Other students spoke about utilizing a similar strategy, where they referred to cases they had encountered in the past for assessing the legitimacy of content or claims presented online.

Yet, many students declared that they felt unsure about what might be considered trustworthy information, since their typical search results contained what they thought to be reliable information mixed in with what they deemed to be information written based on personal opinions, as depicted by one student’s comments:

I have found some websites where...I really want to be like “oh wow this is really good information.” And some of the things they have said are so outlandish I’m like “oh this must be written by...somebody who isn’t truly involved in the industry.” And then [the content] has a veterinarian as an author...it’s discouraging.

S1661

Another student noted that with animal health content on the internet:

There’s so much information and so much different information. Especially in the vet field there’s a lot of different institutes of thought and not a lot of things agree all the time when you’re trying to get information.

S1245

These comments highlighted that even within Web-based resources affiliated with the veterinary profession, views presented could differ vastly.

#### Handling Conflicting Information

From the students’ perspective, conflicting ideas presented by multiple internet resources affiliated with the veterinary profession and assumed to be reputable appeared to complicate their attempts at evaluating information quality.

When discussing information quality and the evaluation criteria students applied, one commonality observed seemed to be the importance students placed on authorship. This was well exemplified by the comments of one student describing her information assessment process:

Looking at who published it, is it actually a veterinarian, is it just someone with a really strong opinion and a platform using the Internet.

S1549

Related to skepticism about authorship, students discussed taking into account the format in which the veterinary messages are presented when evaluating information. In particular, students described blogs as less reliable sources for information:

I try to avoid blogs, and that kind of stuff and try to find sources like some veterinary hospitals have a lot of great resources online...government web pages. Things like that, I try to stick with if I'm actually looking for valid information.

S1660

Accentuating the apparent caution students applied when reading blogs, some students expressed a belief that authors of blogs may be writing to publish opinions, as depicted by one participant:

A lot of blogs and websites where they write for the sake of an outlet.

S1658

However, one student would consider blogs as an information source depending on authorship, stating the following:

I know there’s some blogs that are written by veterinarians...So if it’s written by someone who seems credible, who has the educational background...it’d be more credible. And is information I’d feel more comfortable trusting.

S1660

Overall, students appeared to consider authorship and whether the author may be a veterinarian, as depicted by one participant:

There are a few websites where I know the veterinarians personally that publish onto them...So if I can I try to look for them because I know a little bit more about that veterinarian.

S1551

Elaborating on authors being veterinarians, nearly all students’ information evaluation criteria involved examining authors’ credentials:

The first thing I do before reading...is look at the author’s signature...‘Cause it’ll say oh, Dr. ______ and it’ll say board-certified veterinary dermatologist. I see that and I’m like okay, whatever he says is probably more weighty than someone else’s comment.

S1557

One student acknowledged the following with animal health information on the internet:

You can’t just take everything you read for granted. You have to...take it with a grain of salt.

S1661

## Discussion

### Principal Findings

Findings of this study offer an understanding of veterinary students’ perceptions about the nature of Web-based animal health information, and how their search experiences may impact the way in which that information is managed. Students in this study clearly indicated that they were overwhelmed by the volume of Web-based animal health information. These findings are consistent with studies of students in human health sciences programs being inundated by increasing amounts of health-related content from the internet [51,52]. The apparent staggering effect from searching the internet for animal health information appears to stem from the students’ search strategies, specifically search engine use. Using search engines for seeking health information produces massive volumes of content for seekers, with 38% in one study reporting feeling overwhelmed as a result [53]. In another study, search engine use for seeking health content among college students found that 19% felt overwhelmed by the search results [54]. Consistent with findings from this study, primarily turning to Google for health content quickly produces unmanageable quantities of information [16,55,56].

Feeling swamped by search engine results may be linked to the students’ frustration and emotional responses to seeking Web-based health information. According to Spink et al [57], “Internet users are often frustrated and emotional during their Web search engine interactions. They wish to engage in an advice-seeking interaction, but may be frustrated by the inability of the system to respond to their personal medical and health needs and concerns for health information” (p. 49). Similarly, veterinary students may experience feelings of frustration from using search engines because these tools may not give precise responses to students’ specific animal health inquiries. In this study, this claim is supported by students’ reports of using search engines during lectures. The circumstance of being in class also highlights the fact that students needed information immediately imposing some degree of urgency, which may contribute to feelings of frustration. During these searches, students entered just 1 or 2 terms into the search engine, generating large volumes of content which did not meet the students’ immediate information needs [58,59]. Understandably, individual students may experience frustration or emotional reactions given the situation—the lecture would be continuing, while the student continued not understanding the materials presented earlier in the same lecture. In addition, the students in this study expressed that they simultaneously tried to manage the information generated from the internet and remain engaged in the ongoing lecture content, causing confusion and potentially increasing that individual’s knowledge gap [60].

As students accepted into a veterinary medical program have robust records of academic achievement [61], becoming aware of one’s knowledge gaps while being unable to quickly ameliorate the situation may further contribute to an individual student’s experiences of frustration. Research on medical students suggests an association between individual anxiety and concerns about mastering knowledge [62]. Given the similarities between medical and veterinary students such as being elite academic performers [63], the latter group presumptively also experiences concerns toward mastering knowledge. Added to this, anxiety related to concerns about academic performance has been documented in veterinary students [60]. Veterinary students’ learning experience may be enhanced and supported by furthering their HIL skills, such that the students feel more confident in their abilities to retrieve and utilize Web-based information.

Students in this study voiced frustration at the time and effort it takes to retrieve relevant Web-based health information and made specific mention of their use and reliance on the Web search engine Google. Among the first-year dental students, research indicates that the reliance on Web search engines such as Google indicates lower levels of EHIL [64]. Other studies of veterinary students have suggested the need to integrate EHIL into curricula [9,28,65], such that graduates will possess adequate research skills compliant with accreditation standards 5 and 12 of the American Veterinary Medical Association that will benefit graduates in either research or clinical practice. The results of this study also support facilitation of Web-based HIL skills. Students’ apparent frustration may be ameliorated if they receive more opportunities to use their existing EHIL capabilities, along with receiving feedback from instructors about their information retrieval strategies [65-67].

Paradoxically, while the students from this study expressed being frustrated, and at times confused in their information retrieval efforts, they discussed in detail their skills in evaluating information reliability. Being skeptical appeared to be a common approach for students when assessing information from the internet, as depicted by mentions of critically appraising Web-based content. Students recognized that their skepticism included questioning their own assumptions about information reliability on the basis of past knowledge, acknowledging that some Web-based research findings may conflict with what they had considered to be pre-existing reliable information. These findings appear consistent with research on fourth- and fifth-year medical students judging medical information from the internet according to their own knowledge, while maintaining awareness of their assumptions about information validity [68].

Searching for information on the internet appears to facilitate students building multiple layers of knowledge. The students in this study discussed how the internet affords a quick resource for students to conduct searches for basic content to facilitate learning of more advanced materials. Learners acquiring information using Web-based inquiry for achieving higher level knowledge has been termed technology-enhanced scaffolding [69]. From research on teaching methods, scaffolding provides an individual learner with just enough knowledge to make progress on his or her own [69,70]. However, Web-based scaffolding appeared to also initiate veterinary students’ circular research behavior. The circular research behavior identified in this study is consistent with previous research, which reported that information-seeking processes depended on how an individual perceived the tasks he or she needed to complete [71]. As suggested by these findings, a veterinary student may face the task of understanding a novel concept presented during a lecture. The student may perceive this learning task as a problem that could be resolved by collecting information, where information potentially offers a positive change in the student’s knowledge [72]. How the student obtains information to solve the perceived problem depends on his or her previous knowledge, which also determines how complex they view the task at hand. Expectedly, the less previous knowledge the student possesses, task complexity increases as the student first needs to assimilate basic ideas for comprehending the more advanced content or *need to know to understand*. In turn, this increases how much information the student will need to acquire for accomplishing the original task.

### Implications

Developing EHIL as part of veterinary curricula may reduce student’s feelings of IO. Having enhanced information skills may allow students to rely less on the Google search engine and potentially conduct more efficient, exhaustive content searches in shorter periods of time. For example, a study by Grant et al [28] involving third-year pharmacy students found statistically significant differences in student search strategy test scores after the students received repeated training in Web-based search methods, where the participants were given both a lecture and demonstration on search strategies. Perhaps having similar EHIL training exercises as part of veterinary curricula will confer similar positive experiences for veterinary students. A *protective factor* conferred by EHIL training was suggested by Chemers et al [72] in a longitudinal study of college students, where positive effects of student experiences including EHIL training were strongly associated with academic performance and coping perceptions on stress, health, and overall satisfaction and commitment to remain in school. Similarly, EHIL training may help to support veterinary students’ academic experience and potential subsequent positive outcomes on stress levels and learning satisfaction—making this an important consideration for curricula design.

Ideal curricular design would integrate advancing veterinary students’ knowledge about access, retrieval, evaluation, and practical application of Web-based content on the basis of tasks. Students also appear to need guidance about the depth and breadth of knowledge required of them throughout the training process. Guidance with search strategies may support student learning to distinguish relevant from irrelevant information when conducting Web searches to clarify course materials or new content. Repeated opportunities for students to learn information literacy skills may optimize how these skills become integrated into student cognition. Previous studies of students in professional human health care programs found ongoing training sessions in information literacy over a school year improved information retrieval skills compared with isolated training events such as single workshops during the school year [28].

### Limitations

Limitations of this study include possible selection bias as there is a chance that students who agreed to be interviewed may have wished to specifically voice that they were less comfortable or proficient in searching for Web-based health information. This study aimed to explore experiences and viewpoints and was not designed for establishing statistical generalizability. Outcomes of this study provide depth of understanding in a previously underexplored phenomena and will be used to guide the development of a quantitative questionnaire for measuring the frequency and distribution of some of the observed phenomena in a larger student population.

### Suggestions for Future Work

Empirical studies to investigate relationships between veterinary student HIO, stress, and the role of the internet are warranted. Studies of veterinary students in Australia and the United States have reported higher levels of perceived stress in veterinary students compared with the general population [39]. Presently, however, few publications exist specifically investigating a relationship between information literacy and students’ perceived stress levels. More research into this area appears to be a rational next step, and a direction for future research.

Students’ views and experiences in searching for Web-based pet health information suggest that large volumes of information and the need for evaluation and processing information are 2 aspects that students are negotiating in their efforts to accumulate meaningful content for their learning and understanding. Given a recent AAVMC report considers health literacy as a professional competency [9], future studies may consider investigating student perspectives about HIL. In particular, research focusing on veterinary student confidence and competence toward their existing levels of information literacy may offer an increased understanding about veterinary students’ educational experiences.

### Conclusions

Veterinary students’ perceived experiences of being overwhelmed after searching the internet for animal health information appears to be a discipline-specific manifestation of HIO. Similar to other health professional students reporting experiences of HIO from internet searches for biomedical content, veterinary students’ HIO may be related to their EHIL skills. The findings of this study point to a need for veterinary curricula designers to consider integrating EHIL skill development throughout study semesters for providing students with strategies to manage information.

## Appendix

Multimedia Appendix 1 Interview question guide.

Multimedia Appendix 2 Thematic analysis code table.

## Abbreviations

AAVMC: Association of American Veterinary Medical Colleges
EHIL: electronic health information literacy
EHR: electronic health record
HIL: health information literacy
HIO: health information overload
IO: information overload
