# Supplementary material for: Students' Experiences of Seeking Web-Based Animal Health Information at the Ontario Veterinary College: Exploratory Qualitative Study
Source: JMIR Med Educ. 2019 Nov 8;5(2):e13795. doi: 10.2196/13795 (PMC6874805; doi:10.2196/13795)
Supplement: Multimedia Appendix 1 [file mededu_v5i2e13795_app1.pdf]

Appendix: Multimedia Appendix I

DVM STUDENT FOCUS GROUP
AN EXPLORATION OF UNDERGRADUATE VETERINARY STUDENTS' EXPERIENCES AND PERSPECTIVES REGARDING ONLINE PET HEALTH INFORMATION

(Turn on recorders)

Introduction (5 minutes, begin at xx)

- Hello and welcome

- Thanks for taking the time to join me today for this discussion. We are very interested about your thoughts and experiences in relation to seeking pet health information on the Internet.

- My name is Nanette Lai and I will be the moderator for today's discussion.

- During our discussion today I hope to get your thoughts regarding pet health information online, how this has differed from your previous learning experiences, and how you think this may change how veterinary medicine is practiced.

- I have invited you here today because you are undergraduate students currently enrolled in the DVM program.

- As you have probably already noticed recorders on the table to record our conversation. The recorders are here to capture everything that is said. Myself and a person employed to transcribe the recordings are the only two people who will listen to the recordings. To protect your confidentiality, your names will not be attached to any part of the transcriptions so please feel free to speak freely. Can I request that you speak up please when you are talking so that your valuable comments are captured on the recording.

- While a focus group methodology cannot assure complete confidentiality because it is essentially a public process, I ask that you do not repeat anything that is said here to others outside of the group. This is because I want everyone here to feel safe and comfortable about sharing their thoughts and making comments. I can assure you that I will hold your identifying information in the strictest of confidence. To that end, any information you do not want publicly disclosed should be withheld at your own discretion

- My role here today is to ask questions, listen, keep the conversation moving, ensure everyone gets involved, and be mindful of the time. We may also choose to write up

some of today's findings from this process for publication. For this purpose, de-identified verbatim quotes may be used.

- I would like to hear from all of you but I respect that everyone will participate in different ways. Some people are more talkative, while others are quieter. I will try to ensure that everyone has a chance to speak today. If at any point I have to interrupt you speaking, it will only be because we are running out of time, it is most definitely not because I am not interested in what you have to say.

- To help with this process, I am going to ask that only one person speak at any given time. It is okay to disagree with each other because there are no right or wrong answers here.

- Please be aware that you have the option to withdraw from this study at any time. If you need to leave today for any reason please do this quietly. However, please note that your contributions cannot be withdrawn in the event that you choose to withdraw from the discussion and leave.

- We have 1.5 hours today and there are a number of topics to discuss. Once we are finished today, we will be having lunch/dinner.

- So that we are not interrupted while we chat, can I please ask that you turn any cell phones or electronic devices off for the duration of our discussion today? Thank you.

- If there are any questions I ask today that are not clear or do not make sense then please ask me to clarify them for you.

- Are there any questions? Okay, let's start.

Icebreaker (5 minutes, begin at xx)

- So let's start by going around the table and introducing ourselves. Tell us your name, the names and species of pets you currently own, and their ages.

- I will start…

Participants' guided discussion of their experience seeking pet health information online (60 minutes, begin at xx)

As I mentioned you are currently students enrolled in one of the four phases in the Doctor of Veterinary Medicine program. I am interested in your experiences seeking pet health

information on the Internet, the reasons behind this practice, how you conducted searches, how these experiences may impact your learning, and how they may impact your future career.

Let's begin by discussing your experience seeking pet health information online. I would like you to take a minute and think about the most recent time you did this, what did you search for?

(Go around the room for responses if there is no spontaneous response from anyone to begin the discussion. NL to invite responses from people who did not respond)

Probes:
What prompted you to seek the information you sought?
How did you assess reliability of the sources?
What did you do with the information?
What part of your search experience do you wish could have happened differently?

What web-based resources do you typically access for pet health information?

(Go around the room for responses if there is no spontaneous response from anyone to begin the discussion. NL to invite responses from people who did not respond)

Probes:
How did you learn of these resources?
What about this resource do you find the most appealing?
What makes this resource useful to you?
What role does social media platforms, such as Facebook or Twitter, play in regards to your seeking pet health information on the Internet?
What other social media platforms have been involved in your searches?

What challenges have you experienced with web-based pet health information?

Probes:
How did you overcome these challenges?
What frustrated you the most?

How has the accessibility of web-based information affected your learning experiences as DVM students?

Probes:
When do you consult web-based information in regards to schoolwork?
How has this strategy worked for you?
What would you maintain?
What would you change?

Appendix: Multimedia Appendix I

Overall, **how would you describe the impact web-based pet health information has had on your training as veterinary students?**

What role(s) do you expect web-based information to play in your future career in veterinary medicine?

Probes:
Which resources do you expect yourself to continue using?
When do you foresee yourself using web-based pet health information as practitioners?
How often do you think you'll be using web-based information in your veterinary career?
How do you think web-based information will impact your interactions with clients?
And your interactions with patients?

Regarding the future of vet medicine and the internet,
What direction(s) do you see web-based pet health information heading?
What does this mean for veterinary medicine in the future?
What roles do you expect future veterinarians such as yourselves to be taking in regards to web-based pet health information?

How would you describe the role of companion animals in your life?

Probes:
How did you acquire your first pet?
Your own pets at home. What role do you think of yourself as taking in relation to them?
How does this impact your internet use for pet health information?
Other peoples' pets.
As a DVM student, what role do you feel you play to these pets?

<u>**Summary**</u> (5 minutes, begin at xx)

We have covered all the topics for discussion today so I will summarize what we have discussed and would like you to let me know if there is anything I have left out.

We began with talking about your experiences seeking pet health information on the Internet. Your responses included….

Next, we talked about how your experiences with web-based pet health information may influence your training as veterinary students. Your responses to this included…

Then, we discussed the future directions you anticipate for yourselves as veterinarians, the industry as a whole, and the role web-based information will play on both of those things.

Finally, we went on to discuss how your views of yourselves in regards to companion animals have impacted how you seek pet health information. Your responses to this included…

Is that a fair summary of what we discussed today?

Is there anything else you would like to add to this summary, have I missed anything?

Participants to complete a short demographics questionnaire (1 minute, begin at xx)

(Hand out demographic questionnaire)

The very final thing for you to do today is to fill in this questionnaire about yourselves. It should only take you a minute to complete. The information you provide on this questionnaire is important to us to help keep track of who took part in this discussion. We do not need your name on these as all responses are anonymous and will be kept confidential.

(NL to collect paper with participants' responses)

Conclusion (1 minute)

Thank you very much for coming today to join in this discussion. This discussion is going to be valuable information for me to better understand how web-based information is sought and utilized by veterinary students.

Please help yourselves to some food and non-alcoholic beverages.
