# Supplementary material for: Students' Experiences of Seeking Web-Based Animal Health Information at the Ontario Veterinary College: Exploratory Qualitative Study
Source: JMIR Med Educ. 2019 Nov 8;5(2):e13795. doi: 10.2196/13795 (PMC6874805; doi:10.2196/13795)
Supplement: Multimedia Appendix 2 [file mededu_v5i2e13795_app2.pdf]

Multimedia Appendix II: Code Table

Theme	Sub-theme	Codes	Definition	Example
The Overwhelming Nature of the Internet	Volume and Type of Online Health Information	Overwhelmed by information	Perceptions of experiences searching on the Internet	*"I sometimes get overwhelmed by the amount of information that is out there"*
				"there's just too much [information] out there
	Processing, Managing and Evaluating Information	Need to know to understand	Desiring pre-requisite knowledge required for comprehending novel ideas or content	*"there is so much more behind it that maybe we don't understand…we have an end point but there's so much back research behind it that you…need to know to understand"*
				"…just background information on a topic that you've got no clue about"
		Reading to process information	Describing encounters with text-based content on the Internet	*"the thought for me reading through blog post upon blog post…I find maybe one word that will lead me to something else…"*
